# The applications of DNA methylation as a biomarker in kidney transplantation: a systematic review

**DOI:** 10.1186/s13148-022-01241-7

**Published:** 2022-02-07

**Authors:** Iacopo Cristoferi, Tommaso Antonio Giacon, Karin Boer, Myrthe van Baardwijk, Flavia Neri, Manuela Campisi, Hendrikus J. A. N. Kimenai, Marian C. Clahsen - van Groningen, Sofia Pavanello, Lucrezia Furian, Robert C. Minnee

**Affiliations:** 1 Division of HPB and Transplant Surgery, Department of Surgery, Erasmus MC, University Medical Center Rotterdam, Doctor Molewaterplein 40, 3015GD Rotterdam, the Netherlands; 2 Department of Pathology and Clinical Bioinformatics, Erasmus MC, University Medical Center Rotterdam, Doctor Molewaterplein 40, 3015GD Rotterdam, the Netherlands; 3 Erasmus MC Transplant Institute, Erasmus MC, University Medical Center Rotterdam, Doctor Molewaterplein 40, 3015GD Rotterdam, the Netherlands; 4 Kidney and Pancreas Transplantation Unit, Department of Surgical, Oncological and Gastroenterological Sciences, Padua University Hospital, Via Giustiniani 2, 35128 Padua, Italy; 5 Occupational Medicine, Department of Cardiac, Thoracic, Vascular Sciences and Public Health, Padua University, Via Giustiniani 2, 35128 Padua, Italy; 6 Environmental and Respiratory Physiology Laboratory, Department of Biomedical Sciences, Padua University, Via Marzolo 3, 35131 Padua, Italy; 7 Institute of Anaesthesia and Intensive Care, Department of Medicine - DIMED, Padua University Hospital, Via Cesare Battisti 267, 35128 Padua, Italy; 8 Division of Nephrology and Transplantation, Department of Internal Medicine, Erasmus MC, University Medical Center Rotterdam, Doctor Molewaterplein 40, 3015GD Rotterdam, The Netherlands; 9 Institute of Experimental Medicine and Systems Biology, RWTH Aachen University, Pauwelsstraße 30, 52074 Aachen, Germany

**Keywords:** Systematic review, Kidney transplantation, DNA methylation, Biomarker, Reperfusion injury

## Abstract

**Background:**

Although kidney transplantation improves patient survival and quality of life, long-term results are hampered by both immune- and non-immune-mediated complications. Current biomarkers of post-transplant complications, such as allograft rejection, chronic renal allograft dysfunction, and cutaneous squamous cell carcinoma, have a suboptimal predictive value. DNA methylation is an epigenetic modification that directly affects gene expression and plays an important role in processes such as ischemia/reperfusion injury, fibrosis, and alloreactive immune response. Novel techniques can quickly assess the DNA methylation status of multiple loci in different cell types, allowing a deep and interesting study of cells’ activity and function. Therefore, DNA methylation has the potential to become an important biomarker for prediction and monitoring in kidney transplantation.

**Purpose of the study:**

The aim of this study was to evaluate the role of DNA methylation as a potential biomarker of graft survival and complications development in kidney transplantation.

**Material and Methods:**

A systematic review of several databases has been conducted. The Newcastle–Ottawa scale and the Jadad scale have been used to assess the risk of bias for observational and randomized studies, respectively.

**Results:**

Twenty articles reporting on DNA methylation as a biomarker for kidney transplantation were included, all using DNA methylation for prediction and monitoring. DNA methylation pattern alterations in cells isolated from different tissues, such as kidney biopsies, urine, and blood, have been associated with ischemia–reperfusion injury and chronic renal allograft dysfunction. These alterations occurred in different and specific loci. DNA methylation status has also proved to be important for immune response modulation, having a crucial role in regulatory T cell definition and activity. Research also focused on a better understanding of the role of this epigenetic modification assessment for regulatory T cells isolation and expansion for future tolerance induction-oriented therapies.

**Conclusions:**

Studies included in this review are heterogeneous in study design, biological samples, and outcome. More coordinated investigations are needed to affirm DNA methylation as a clinically relevant biomarker important for prevention, monitoring, and intervention.

**Supplementary Information:**

The online version contains supplementary material available at 10.1186/s13148-022-01241-7.

## Background

Kidney transplantation is the treatment of choice for patients undergoing end-stage renal failure [1] and improves survival and quality of life. A clear amelioration in short-term outcomes has been observed in the last decades, while a proportional improvement in long-term results is still missing because of the immune- and non-immune-mediated complications that affect these outcomes [2–8]. Efforts have been made to improve preventive measures and optimize treatment. Along with this, the identification of patients with a higher risk of post-transplant complications is of great importance. Current biomarkers of post-transplant complications and survival include epitope mismatch [9] and anti-HLA antibodies [10]. Their predictive value is suboptimal, raising the need to explore novel approaches for the management of transplant patients [11–13].

Epigenetic modifications get a lot of interest as a novel biomarker in transplantation. These modifications are reversible changes to the genome that occur without any alteration in the DNA sequence. The three main epigenetic modifications are histone modification, DNA methylation, and nucleosome positioning [14]. DNA methylation consists in the formation of a covalent bond between a methyl group and a cytosine almost exclusively in the context of cytosine-phosphate-guanine (CpG) dinucleotides, often clustered in regions called CpG islands [15] that are associated with about 60% of human genes promoters [16]. DNA methylation is generally associated with gene silencing, primarily affecting transcription [15]. Epigenetic mechanisms play an important role in multiple biological events involved in post-transplant complications development, such as the alloreactive immune response [17–21], ischemia/reperfusion injury (IRI) [22–24], and kidney graft fibrosis [7, 25–30]. DNA methylation assessment of specific loci is also crucial for the evaluation of biological or epigenetic age (DNAmAge) using epigenetic clocks [31–33].

Considering the central role that big data analysis is having in every research field and the new methylation-wide assessment technologies that have been developed, DNA methylation has the potential to become an important biomarker for prediction and monitoring in kidney transplantation, and its use could become pivotal for the development of new therapeutic strategies [7, 34, 35]. Therefore, we performed a systematic review to evaluate the status of research concerning the role of DNA methylation as a biomarker in kidney transplantation.

## Materials and methods

This systematic review was performed according to the guidelines for observational studies as described in the Preferred Reporting Items for Systematic Reviews and Meta-Analyses (PRISMA) statement [36, 37]. The data extraction and results exposition of this review have been organized into two major topics: I ischemia–reperfusion injury, fibrosis, and long-term complications-related studies and II immune response modulation-related studies.

### Search strategy

With the help of a clinical librarian, we searched EMBASE, Medline ALL Ovid, Web of Science, Cochrane CENTRAL Register of Trials, and Google Scholar databases. The search terms for the other databases have been created starting from the EMBASE database search. The search included the following terms: DNA methylation, hypermethylation, hypomethylation, demethylation combined with kidney, renal transplantation, graft, allograft, allotransplantation, fibrosis, recipient, failure, reperfusion, and insufficiency. The databases have been searched from inception to September 30, 2021. For all the articles reaching the full-text-reading phase of the selection, references have been manually checked. Detailed search strategies are included in Additional file 1: Table S1.

### Study selection

The studies were initially reviewed, screening title and abstract, by two independent reviewers (IC and TAG). The following inclusion criteria were applied: original articles (not reviews, editorials, or conference abstracts); English language; working on human samples; study focused on DNA methylation in kidney donors or kidney transplant recipients; at least one DNA methylation assessment performed. No restrictions have been used for study designs, population characteristics, and the number of included subjects. Important exclusion criteria have been used: not focusing on transplantation; focusing on general transplantation or combined transplantation with no possibility to extrapolate kidney-specific data; working only with samples of animal origin; not assessing DNA methylation. Disagreements were discussed between both reviewers and, when necessary, with a third party (RCM).

### Risk of bias assessment

For non-randomized trials, the Newcastle–Ottawa scale [38, 39] has been used to assess the risk of bias. For clinical trials, the expanded six-point version of the Jadad scale [40] has been used to assess appropriate randomization, blinding, and management of withdrawals and dropouts.

Risk of bias has been assessed by two independent reviewers (IC and TAG), and disagreements were discussed between them and, when necessary, with a third party (RCM).

### Data collection and extraction

A data extraction sheet has been developed, and the following features have been extracted from each study: research group, year of publication, country, study design, study’s aim, study population, results, sample tissue, extent of the methylation assessment, bisulfite conversion, methylation assessment method, methylation outcome, statistical tests, and statistical thresholds.

## Results

**Fig. 1:**
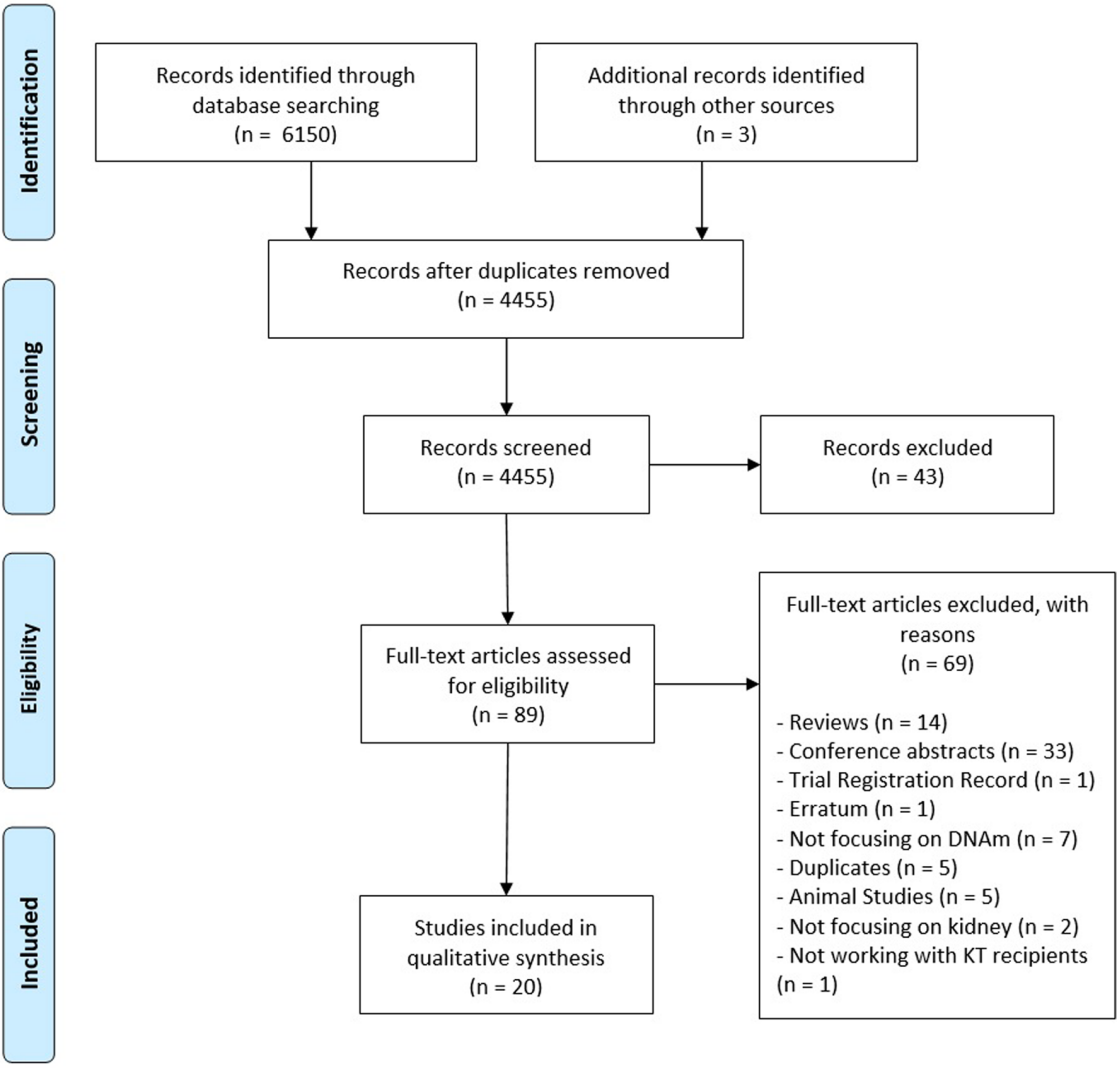
Flow diagram of the systematic literature search

**Fig. 2:**
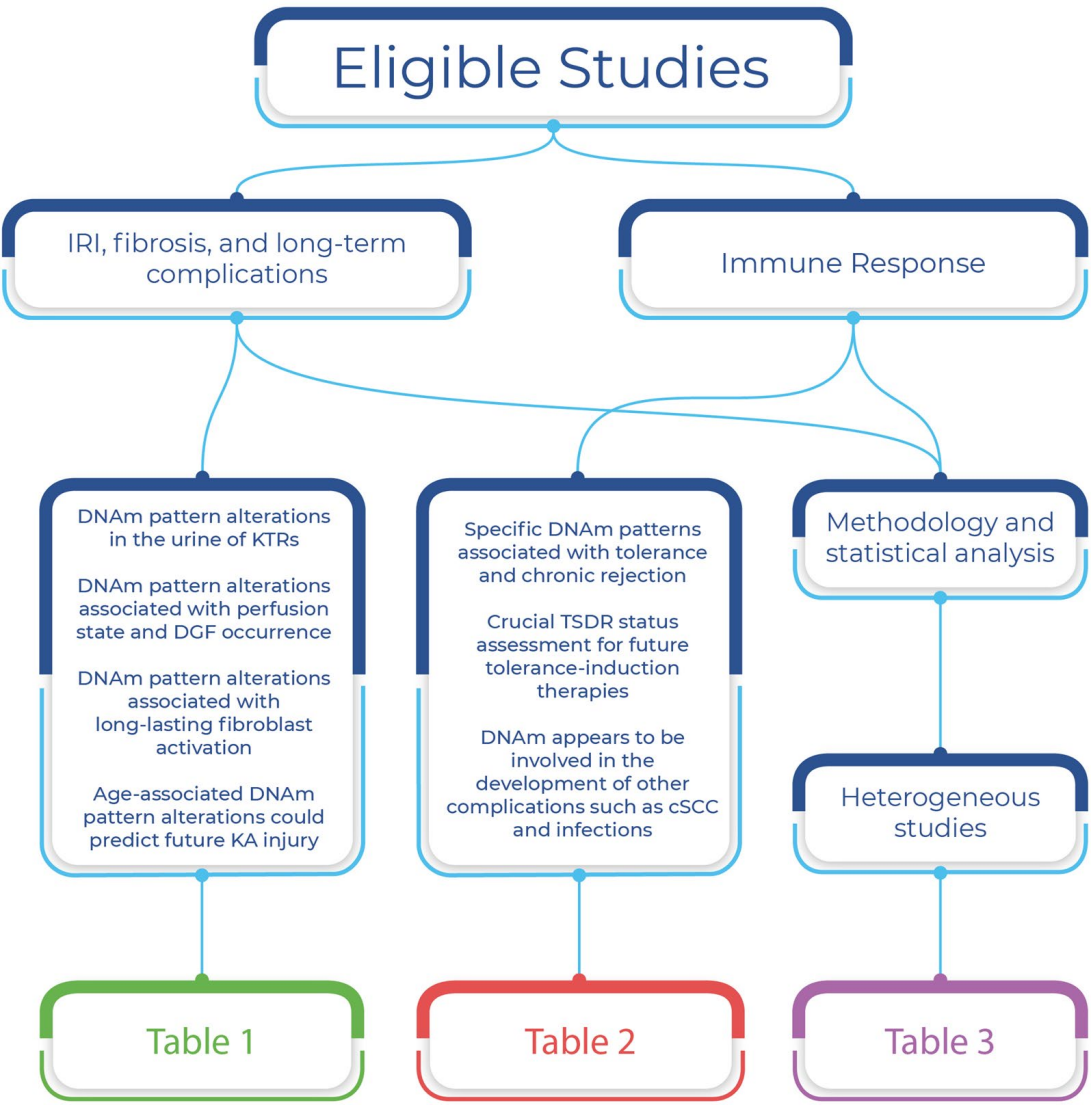
Overview of the summarization strategy. Abbreviations: cSCC, Cutaneous squamous cell carcinoma; DGF, Delayed graft function; DNAm, DNA methylation; KA, Kidney allograft; KTR, Kidney transplant recipient; IRI, Ischemia–reperfusion injury; TSDR, Treg-specific demethylated region

A total number of 4455 potentially relevant studies were identified. Figure 1 presents the PRISMA flow diagram. Twenty studies met the inclusion criteria and were included in the qualitative synthesis. The characteristics of the included studies are summarized in two tables (Tables 1, 2) divided into the two major topics: I ischemia–reperfusion injury, fibrosis, and long-term complications-related studies; II immune response modulation-related studies. The methodology, the statistical analysis, and the identified candidate genes of the included studies are summarized in Table 3. Figure 2 represents an overview of the summarization strategy and the main findings of this systematic review. The included studies were conducted between 2006 and 2021 in ten different countries. Study sample size ranged from 9 to 188, with a mean size of 72.3 (in two studies, only the number of biopsies was provided). Fourteen studies worked on blood samples, five studies used kidney biopsies, and urine has been used by a single study. For what concerns DNA methylation analysis design, six studies performed only genome-wide analysis, 11 studies performed only candidate genes analysis, among which eight studies investigated the methylation status of the Treg-specific demethylated region (TSDR), while three studies performed epigenome-wide analysis as a first step and then investigated the methylation status of candidate genes.

**Table 1:** Data extraction chart for IRI, fibrosis, and long-term complications-related studies

References	Country	Study design	Study’s aim	Study population	Results
Mehta et al. [41]	USA	CS OBS	To find changes in urine epigenetics suitable as biomarkers in early KT injury and repair	*N* = 88 KTRs cohort: 23 patients (13 DD, 10 LD) on day 2 HC: 65	Differential DNAm in urine of loci like *CALCA* promoter of KTRs compared to HC and DDs compared to LDs. No significant correlation between patients undergoing ATN vs. AR and between patients undergoing DGF vs. HCs
Bontha et al. [42]	USA	CS OBS	To understand oxidative stress and inflammatory setting trigger changes in DNAm patterns of the KA leading to fibrosis development and graft dysfunction	*N* = 95 Patients: 95 KTR of DD grafts, 99 biopsies 59 Post-KT (30 IFTA, 29 NFA) 40 Pre-KT (20 IFTA, 20 NFA)	A relationship between DNAm pattern alterations and IFTA has been found, with specific patterns involving fibrosis-related pathways, mostly acting on transcription factors and homeobox genes
McGuinness et al. [43]	UK	R OBS	To identify molecular signatures associated with DGF, to adjust for the effects of IRI, and to validate by comparison with publicly available data sets	*N* = 55 55 KTRs: PPPs from DD 23 extreme DGF phenotype or IGF phenotype	Specific transcript promoter’s differential DNAm upon perfusion state and DGF occurrence has been found, identifying molecular signatures associated with DGF
Heylen et al. [44]	Belgium	CS OBS	To investigate whether ischemia induces DNA HrM and contributes to chronic injury	*N* = ? Biopsies, 3 cohorts 13 PPPs + 2 × 5 in a subgroup at 3 or 12 months; 82 biopsies immediately before KT; 46 postreperfusion biopsies; Validation cohort: 10 postreperfusion biopsies	DNAm pattern alterations involving genes suppressing kidney injury and fibrosis were found, linking ischemia at the time of KT with progressive chronic allograft injury at 1 year after KT. Ischemia seemed to reduce TET enzymes activity
Heylen et al. [45]	Belgium	CS OBS	To understand kidney-associated DNAm changes in the context of aging, trying to find out which specific genes are affected	*N* = ? Biopsies Discovery cohort: 95 prior to KT (82 BDDs, 13 LDs) Validation cohort: 67 immediately after KT and reperfusion (58 BDDs, 9 LDs)	Age-associated changes in DNAm at the time of KT predicted future injury of the KA and epigenetic renal aging is implicated in progressive fibrosis in both the glomerulus and the interstitium. No association was found between the methylation patterns, arteriosclerosis, and tubular atrophy
Schaenman et al. [46]	USA	P OBS	To prove the potential benefit of DNAmAge analysis in the context of KT, trying to associate DNAmAge and infection occurrence	*N* = 60 Old cohort: 24 Young cohort: 36	DNAmAge analysis holds promise for improving clinical outcomes and has been associated with post-KT infections. DNAmAge may be higher or lower than chronological age

BDD, Brain-dead donor; CALCA, Calcitonin-related polypeptide alpha; CS, Cross-sectional; DNAm, DNA methylation; DNAmAge, Epigenetic age; HC, Healthy controls; HrM, Hypermethylation/ed; IFTA, Interstitial fibrosis and tubular atrophy; IGF, Immediate graft function; KA, Kidney allograft; KT, Kidney transplant/transplantation; KTR, Kidney transplant recipient; LD, Living donor; NFA, stable functioning allograft with no or minimal IFTA; OBS, Observational; P, Prospective; PPP, paired preperfusion/postperfusion kidney biopsy; R, Retrospective

**Table 2:** Data extraction for immune response modulation-related studies

References	Country	Study design	Study’s aim	Study population	Results
Bestard et al. [47]	Spain/Germany	R OBS	To confirm that FOXP3-expressing T cells in SCR patients are Treg cells and investigate whether the benefit from FOXP3^+^ Treg cells infiltrates in SCR patients is valid in the long term	*N* = 105 25 KTRs with SCR with FOXP3^+^ Treg cells 12 SCR KTRs without FOXP3^+^ Treg cells 68 SCR^−^ KTRs	Intragraft FOXP3^+^ T cells in SCR patients positively correlated with *FOXP3* HoM at TSDR. Worse 5-year GF for SCR patients without FOXP3^+^ T cells infiltrate compared with SCR patients with infiltrate. Patients with SCR and IFTA had the same graft outcome as biopsy-negative patients if a FOXP3^+^ T cells infiltrate was present
Bouvy et al. [48]	Netherlands	P OBS	To assess how depleting and non-depleting induction therapies influence the mechanism of Treg cells homeostasis in KTRs	*N* = 33 33 KTRs: 15 rATG treatment 18 Basiliximab treatment	Repopulation of Treg after rATG and basiliximab therapy is the result of homeostatic proliferation and not of thymopoiesis. With both induction therapies, Treg cells could inhibit allospecific T cells. Only after rATG therapy increased proportions of Helios^−^ methylated FOXP3 Treg cells could be found
Braza et al. [49]	France/Germany	CS OBS	To characterize Tregs in TOL patients	*N* = 80 15 HV 13 TOL 33 STA 19 CR	TOL patients mobilized an array of potentially suppressive cells, both regulatory B and T cells. These patients had potent CD4^+^CD45RA^−^FOXP3^hi^ memory Treg cells with a specific TSDR HoM pattern
Sherston et al. [50]	UK/Germany	CS OBS	To determine whether PBMCs DNAm remains stable over time and identify TSDR-demethylated cells in a cohort of long-term KTRs survivors with and without cSCC	*N* = 58 32 cSCC KTRs 26 cSCC^−^ KTRs	The immune phenotype proved to be stable and may be a valuable biomarker for cSCC identification, especially TSDR HoM lymphocytes, since this study proved that elevated circulating Treg cells levels have been associated with a history of cSCC in both immunosuppressed and non-immunosuppressed individuals
Boer et al. [51]	Netherlands	R OBS	To examine the influence of variations in DNAm of IFNγ and PD1 in different CD8 + T cell subsets on AR	*N* = 40 CMV/DNAm assessment: 15 CMV^+^ HD 15 CMV^−^ HD DNAm /AR KTRs from CMV^−^ HD: 5 biopsy-proven AR KTRs 5 AR^−^ KTRs	DNAm of IFNγ and PD1 increased at 3 months after KT in memory CD8 + T cells in KTRs, irrespectively of rejection occurrence, indicating that the KT procedure contributed to these variations. At 12 months, no difference was found
Trojan et al. [52]	Germany	CS OBS	To assess whether KTRs with stable GF possess a certain pattern of Treg cells in the blood that is different from the one of HCs	*N* = 188 Patients: 136 KTR HC: 52	KTRs with stable GF possessed IFNγ^+^ and IFNγ^−^ Treg cells with stable and unstable FOXP3 expression in the blood coexpressing CD28, HLA-DR, CTLA4, CXCR3, Lselectin, TGFβ, perforin, and FasL that might contribute to the establishment and maintenance of good long-term GF
Trojan et al. [53]	Germany	P OBS	To investigate the absolute cell counts of IFNγ^+^ Treg cells subsets in KTRs with good long-term GF, whether they are mainly tTreg or pTreg and whether their expression of FOXP3 is stable or transient in order to offer clues about *in vivo* vs. *in vitro* Treg expansion for therapeutic purposes	*N* = 188 First sample: 136 KTR 52 HC 3 months sample: 59 KTR 6 months sample: 11 KTR	KTRs with good long-term GF had comparable levels of IFNγ^+^ Treg cells to HCs. Their IFNγ^+^ and IFNγ^−^ Treg cells subsets showed stable and transient FOXP3 expression. Their IFNγ^+^ and IFNγ^−^ Treg cells were more frequently HrM compared with HCs and their levels of Treg cells subsets with stable and transient FOXP3 expression were increased compared to HCs. No association was found between the levels of methylation and kidney function or previous episodes of AR or infections
Alvarez Salazar et al. [54]	Mexico	INT	To analyze the impact of long-term therapy with BLT or CsA on the phenotype, suppressive function, and the epigenetic status of the *FOXP3* TSDR from peripheral Tregs of STA KTRs	*N* = 44 Patients: 35 24 BLT-treated 11 CsA-treated HC: 9	Circulating Tregs are not solely responsible for tolerance in long-term patients. Only BLT-treated patients have increased cellular population showing *FOXP3* HoM that could promote tolerance. Tregs from transplanted patients showed significantly reduced suppressive capacity
Peters et al. [55]	Netherlands	R OBS	To identify KTRs at risk for de novo post-KT cSCC by studying genome-wide DNAm of T cells	*N* = 54 27 cSCC 27 non-cSCC	16 DMRs between patients with future cSCC and HC in regulatory genomic regions, 5 of which were stable after transplantation and could have a lasting effect on post-KT cSCC development
Peters et al. [56]	Netherlands	R OBS	To prove that functional differences in circulating T cells represent risk factors in the development of a *de novo* post-KT cSCC	*N* = 120 Pre-cSCC: 19 cSCC 19 non-cSCC During cSCC 45 cSCC 37 cSCC	Different DNAm, transcriptional regulation, and protein expression of *SERPINB9* between cSCC and non-cSCC patients, identifying a novel risk factor for post-KT cSCC development and providing mechanistic insight into the role of circulating T cells in cSCC development
Cortés-Hernández et al. [57]	Mexico	INT	To clarify whether an *ex vivo* expansion of Tregs from patients who underwent long-term immunosuppression may be feasible to be used in their treatment	*N* = 9 Patients: BSX- and BLT-treated under maintenance therapy undergoing *ex vivo* expansion of Tregs Control: HC from the blood bank	Expansion of Tregs from long-term BLT-treated patients displayed high suppressive activity after 4 weeks. However, the detected lower level of TSDR HoM may require the use of epigenetic modifying agents to stabilize FOXP3 expression to be considered as a valid treatment
Zhu et al. [58]	China	R OBS	To understand if DNAm patterns are modified after KT and if this alteration could influence the fate of KAs	*N* = 23 Graft dysfunction cohort: ? Graft stable cohort: ? 13 HC	Methylation modification occurred after KT, involving the mTOR signaling pathway. Higher activity in the case of AR-induced AD
Soyoz et al. [59]	Turkey	P OBS	To understand the expression and epigenetic modifications of IL-2, IL-4, and IFNγ in CD4^+^ T cells of KTRs undergoing AR	*N* = 25 13 STA 6 AR 6 GD	Increased expression of IFNγ with changes in the methylation status of the + 128 CpG in CD4^+^ T cells of KTRs undergoing AR
Rodriguez et al. [60]	Spain/UK	CS OBS	To analyze DNAm patterns in KTRs with CR and TOL	*N* = 36 7 HC 9 TOL 6 CR 7 MO 7 TT	DNA methylation changes associated with transplant outcome and TOL associated with different DNAm patterns in genes related to B and T cell function. Patients undergoing CR displayed DNAm pattern alterations on genes related to the ubiquitination pathways

AD, Allograft dysfunction; AR, Acute Rejection; BLT, Belatacept; BSX, Basiliximab; CMV, Cytomegalovirus; CpG, Cytosine-phosphate-guanine site; CR, Chronic rejection; CS, Cross-sectional; CsA, Cyclosporine A; cSCC, Cutaneous squamous-cell carcinoma; CTLA4, Cytotoxic T-lymphocyte-associated protein 4; CXCR3, CXC-motive-chemokine-receptor 3 (CD183); DMRs, Differentially methylated regions; DNAm, DNA methylation; FOXP3, Forkhead box P3 or scurfin; FasL, Fas ligand; GD, Graft dysfunction; GF, Graft function; HC, Healthy controls; HD, Healthy donors; HLA-DR, Human leukocyte antigen – DR isotype; HoM, Hypomethylation/ed; HV, Healthy volunteers; IFNγ, Interferon γ; IFTA, Interstitial fibrosis and tubular atrophy; IL, Interleukin; INT, Interventional; iTreg, Induced regulatory T cell; KA, Kidney allograft; KT, Kidney transplant/transplantation; KTR, Kidney transplant recipient; Lselectin, L-selectin (CD62L); MO, stable patients with only low-dose prednisone therapy; mTOR, mammalian target of rapamycin; OBS, observational; P, Prospective; PBMC, Peripheral blood mononuclear cell; PD1, Programmed cell death protein 1; pTreg, Peripherally induced regulatory T cells; R, Retrospective; rATG, Rabbit anti-thymocyte globulin; SCR, Subclinical cellular rejection; SERPINB9, Serpin Family B Member 9; STA, Stable function; TGF-β, Transforming growth factor β; TOL, Operationally tolerant/operational tolerance; Treg cells, Regulatory T cells; TSDR, Treg-specific demethylated region; TT, stable patients on standard triple therapy; tTreg, Thymus-derived regulatory T cells

**Table 3:** Overview of methodology and statistical analysis

References	Sample tissue	Epigenome-wide, candidate genes, or TSDR methylation status	Bisulfite conversion	Method	Methylation outcome	Statistical tests	Statistical thresholds
*IRI, fibrosis, and long-term complications-related studies*							
Mehta et al. [41]	Urine	Candidate Genes (*CALCA*)	In-house [41]	qPCR (TaqMan, primer: designed for the *CALCA* gene locus)	Target Gene/β-actin*1000	Student’s *t*-test, ANOVA, Wilcoxon rank-sum test, Kruskal–Wallis test to compare mean *CALCA* values among the study groups, as appropriate	Not Provided
Bontha et al. [42]	Kidney Biopsy	Epigenome-Wide	EZ DNA methylation kit (Zymo Research)	Infinium HumanMethylation450 BeadChip (Illumina)	β and M values for statistical analysis, no value presented	Moderated Student’s *t*-test BY-corrected to compare DNAm levels between IFTA and NFA patients, PCA and average linkage hierarchical clustering of DNAm, GE, and miRNA integrated datasets	FDR < 0.01
McGuinness et al. [43]	Kidney Biopsy	Epigenome-Wide	EZ DNA methylation kit (Zymo Research)	Whole Genome Bisulfite Sequencing (EpiGnome Methyl‐Seq kit (Illumina) to generate libraries and NextSeq500 (Illumina) for sequencing)	Methylated cytosines within CpG dinucleotides (mCpGs)	Kruskal–Wallis test with FDR correction to compare differences in CpG DNAm status of DGF-specific transcripts among the study groups	FDR < 0.05
Heylen et al. [44]	Kidney Biopsy	Epigenome-Wide	EZ DNA methylation kit (Zymo Research)	Infinium MethylationEPIC BeadChip (Illumina) Infinium HumanMethylation450 BeadChip (Illumina)	β and M values for statistical analysis, methylation percentage for visualization	Wilcoxon rank-sum test to compare pre- versus post- ischemia DNAm levels, linear regression to examine the effect of CIT on DNAm of all CpGs in the pre-KT cohort, paired Student’s *t*-test to examine the effect of CIT on DNAm of CpGs grouped per CGI in the longitudinal cohort, linear mixed model to examine the effect of CIT on DNAm of CpGs grouped per CGI in the pre-KT cohort, binomial tests to compare HrM and HoM events	FDR < 0.05
Heylen et al. [45]	Kidney Biopsy	Epigenome-Wide	EZ DNA methylation kit (Zymo Research)	Infinium MethylationEPIC BeadChip (Illumina) Infinium HumanMethylation450 BeadChip (Illumina)	M values for statistical analysis, coefficients based on β values for visualization	Linear regression to examine the effect of age on DNAm, binomial tests to compare HrM and HoM events, linear regression to associate DNAm levels of all age-associated CpGs to histology scores, logistic regression to associated DNAm levels of all age-associated CpGs to reduced allograft function	FDR < 0.05
Schaenman et al. [46]	Blood sample (PBMCs)	Epigenome-Wide	EZ DNA methylation kit (Zymo Research)	Infinium MethylationEPIC BeadChip (Illumina)	M values and DNAmAge (Horvath method)	Kaplan–Meier analysis for time-dependent analyses of infection or rejection in relation to DNAmAge, with statistical Gray’s test to evaluate hypotheses of equality of cumulative incidence functions between study groups	*p* < 0.05
*Immune response modulation-related studies*							
Bestard et al. [47]	Kidney Biopsy	TSDR methylation status	According to Wieczorek et al. [61]	qPCR (primers according to Wieczorek et al. [61])	Methylation percentage	One-way ANOVA, *t*-test, Kruskal–Wallis test, or Mann–Whitney *U*-test to compare different study groups, as appropriate	*p* < 0.05
Bouvy et al. [48]	Blood sample (PBMCs)	TSDR methylation status	EZ DNA methylation kit (Zymo Research)	qPCR (TaqMan, primers according to Wieczorek et al. [61])	Methylation percentage	Kruskal–Wallis test (with Dunn’s multiple comparison test) to compare multiple groups and Mann–Whitney *U*-test to compare different groups or time-points	*p* < 0.05
Braza et al. [49]	Blood sample (PBMCs)	TSDR methylation status	According to Wieczorek et al. [61]	qPCR (primers according to Wieczorek et al. [61])	Methylation percentage	Kruskal–Wallis test to compare multiple groups	*p* < 0.05 *p* < 0.01 *p* < 0.001
Sherston et al. [50]	Blood sample (PBMCs)	TSDR methylation status	EpiTect Bisulfite Kit (Qiagen)	qPCR	Methylation percentage	Not Provided	*p* < 0.05
Boer et al. [51]	Blood sample (PBMCs)	Candidate-genes (*PD1* and *IFNγ*)	EZ DNA methylation kit (Zymo Research)	PCR amplification and pyrosequencing (primers designed for h *PD1* and *IFNγ* genes loci)	Methylation percentage	Student’s *t*-test, ANOVA, or Mann–Whitney *U*-test, as appropriate, to compare different groups; linear mixed-effects model to determine differences after KT over time between rejectors and non-rejectors	*p* < 0.05
Trojan et al. [52]	Blood sample (PBLs)	TSDR methylation status	EZ DNA methylation kit (Zymo Research)	qPCR (primers: Human Foxp3 Methylation Panel, EpigenDx)	Methylation percentage	ANOVA, Wilcoxon test, Mann–Whitney *U*-test, and Spearman rank correlation test to compare different study groups, as appropriate	FDR < 0.01
Trojan et al. [53]	Blood sample (PBLs)	TSDR methylation status	EZ DNA methylation kit (Zymo Research)	qPCR (primers: Human Foxp3 Methylation Panel, EpigenDx)	Methylation percentage	Wilcoxon test or Mann–Whitney *U*-test to compare different study groups, as appropriate	FDR < 0.01
Alvarez Salazar et al. [54]	Blood sample (PBMCs)	TSDR methylation status	EZ DNA methylation kit (Zymo Research)	PCR amplification and sequencing (primers’ sequence reported)	Methylation percentage	Kruskal–Wallis test to compare more than 2 different study groups and Mann–Whitney *U*-test to compare 2 different study groups	*p* < 0.05
Peters et al. [55]	Blood sample (PBMCs)	Epigenome-wide and Candidate Genes validation (*RNF180* and *ZNF502*)	EZ DNA methylation kit (Zymo Research)	Epigenome-wide Infinium HumanMethylation450 BeadChip (Illumina) Candidate Genes confirmation PCR amplification and pyrosequencing (primers designed for the *RNF180* and *ZNF502* genes loci)	Β values	Linear mixed-effect model to identify DNAm differences between groups, paired Wilcoxon test to compare DNAm levels pre- and post-KT, Mann–Whitney *U*-test to compare different study groups, and Spearman’s rank correlation coefficient to compare the results of the whole epigenome analysis and the pyrosequencing of candidate genes	*p* < 0.05 FDR < 0.05
Peters et al. [56]	Blood sample (T cells)	Epigenome-wide and Candidate Genes validation (*SERPINB9* and *VTRNA2-1*)	EZ DNA methylation kit (Zymo Research)	Epigenome-wide Infinium HumanMethylation450 BeadChip (Illumina) Candidate Genes confirmation PCR amplification and pyrosequencing (primers designed for the *SERPINB9* and *VTRNA2-1* loci)	Β values	Linear mixed-effect model to identify DNAm differences between groups, paired Wilcoxon test to compare DNAm levels before and after transplantation, Mann–Whitney *U*-test to compare different study groups, Spearman’s rank correlation coefficient to compare the results of the whole epigenome analysis and the pyrosequencing of candidate genes	*p* < 0.05 FDR < 0.05
Cortés-Hernández et al. [57]	Blood sample (PBMCs)	TSDR methylation status	EZ DNA methylation kit (Zymo Research)	PCR amplification and Sanger sequencing (primers’ sequence reported)	Methylation percentage	Student’s *t*-test, Wilcoxon rank-sum test, or Mann–Whitney *U*-test to compare different study groups, as appropriate, and Kruskal–Wallis test or one-way ANOVA to compare more than 2 different study groups, as appropriate	*p* < 0.05
Zhu et al. [58]	Blood sample (PBMCs)	Epigenome-Wide and Candidate-genes (*RUNX3, DDIT4, PTEN,* and *FOXP3*)	EpiTect Bisulfite Kit (Qiagen)	Epigenome-wide Infinium HumanMethylation450 BeadChip (Illumina) Candidate-genes PCR and Next-Generation Sequencing (primers designed for the *RUNX3, DDIT4, PTEN,* and *FOXP3* genes loci)	Methylation percentage	Wilcoxon rank-sum test and array-related software packages to determine methylation-variable positions	FDR < 0.05
Soyoz et al. [59]	Blood Sample (PBLs)	Candidate-genes (*IL-2* and *IFN-γ*)	EpiTect Bisulfite Kit (Qiagen)	qPCR and Pyrosequencing (primers designed for the *IL-2* and *IFN-γ* genes loci)	Indicated as increased or decreased	Not provided	Not provided
Rodriguez et al. [60]	Blood sample (PBMCs)	Epigenome-Wide	EZ DNA methylation kit (Zymo Research)	Infinium MethylationEPIC BeadChip (Illumina)	M values and Β values	Mann–Whitney *U*-test to compare different study groups	*p* < 0.05 FDR < 0.05

ANOVA, Analysis of variance; BY, Benjamini–Yekutieli; CALCA, Calcitonin Related Polypeptide Alpha; CGI, CpG island; CIT, Cold ischemia time; CpG, Cytosine-phosphate-guanine site; DDIT, DNA Damage Inducible Transcript; DGF, Delayed graft function; DNAm, DNA methylation; DNAmAge, Epigenetic age; FDR, False discovery rate; FOXP3, Forkhead box P3 or scurfin; GE, Gene expression; HoM, Hypomethylation; HrM, Hypermethylation; IFNγ, Interferon γ; IFTA, Interstitial fibrosis and tubular atrophy; IL, Interleukin; KT, Kidney transplantation; Lselectin, L-selectin (CD62L); NFA, stable functioning allograft with no or minimal IFTA; PBL, Peripheral blood lymphocytes; PBMC, Peripheral blood mononuclear cell; PCA, Principal component analysis; PCR, Polymerase chain reaction; PD1, Programmed cell death protein 1; PTEN, Phosphatase and tensin homolog; qPCR, Quantitative real-time PCR; RNF, Ring Finger Protein; RUNX, RUNX Family Transcription Factor; SERPINB9, Serpin Family B Member 9; TSDR, Treg-specific demethylated region; VTRNA, Vault RNA; ZNF, Zinc Finger Protein

### I. Ischemia–reperfusion injury, fibrosis, and long-term complications

A total of six studies focused on IRI, fibrosis, and long-term complications; one study analyzed urine samples, four studies analyzed kidney biopsies, and one study analyzed blood samples. Detailed information on the individual studies is displayed in Tables 1 and 3. Briefly, aberrant hypermethylation of calcitonin-related polypeptide alpha (*CALCA*) gene in urine samples was significantly more likely to occur in kidney transplant recipients compared to healthy individuals [41]. Four studies investigated potential associations between the methylation state of different loci in kidney transplant biopsies and various clinical conditions that may occur at different time points after transplantation. In the first study, DNA methylation pattern alterations have been associated with early clinical conditions such as delayed graft function (DGF) [43]. The other three studies investigated DNA methylation in relation to ischemia, aging (evaluating DNAmAge), and fibrosis development to show relationships between these phenomena and the development of long-term complications [42, 44, 45]. The final more recent study investigated DNAmAge in peripheral blood mononuclear cells (PBMCs) samples and showed associations with post-transplant infection occurrence [46].

### II. Immune response modulation

Fourteen studies reported on DNA methylation involved in immune-response modulation after kidney transplantation; one analyzed kidney biopsies, the other 13 focused on blood samples. Detailed information on the individual studies is displayed in Tables 2 and 3. Various T cell populations are involved in the allospecific immune response. Cytotoxic CD8^+^ T cells account for most of the adaptive immune response against the graft. Antigen-naïve CD8^+^ T cells are characterized by high methylation of effector genes, which are then demethylated after antigen recognition triggering effector genes expression and, consequently, the immune response activation. Among CD4^+^ T cells, regulatory T (Treg) cells are a subset of cells constitutively expressing high levels of the transcription factor *FOXP3* along with the IL-2 receptor *α* chain CD25. These cells are crucial in the process of acceptance/tolerance of an allograft, considering their ability to suppress immune activation in an antigen-specific manner [62]. It is possible to identify stable Treg cells by measuring the methylation status of the TSDR as a demethylated TSDR characterizes Treg cells, while this region is methylated in other cells. Among the included studies, eight studies investigated DNA methylation patterns in Treg cells associated with different clinical conditions related to kidney transplantation [51], such as subclinical rejection [47], tolerance [49, 60], stable graft function [52, 53], acute rejection [58, 59], and chronic rejection [60]. Three studies investigated the feasibility of Treg cells *ex vivo* expansion for therapeutic purposes in patients undergoing immunosuppressive treatment [48, 54, 57]. The final three studies investigated DNA methylation patterns associated with the development of cutaneous squamous-cell carcinoma (cSCC) [50, 55, 56], a long-term complications of kidney transplantation that is related to the immunosuppressive regime that kidney transplant recipients must follow.

### Quality of evidence

According to the Newcastle–Ottawa scale, the quality of all studies was considered moderate to good. The Newcastle–Ottawa scale assessment can be found in Additional files 2–4: Tables S2–S4. The overall Jadad score is good for randomized studies (Additional file 5: Table S5).

### Purpose of DNA methylation analysis in the included studies

In Table 4, the included studies have been categorized into prediction, monitoring, and decision-making/intervention to assess for which purpose DNA methylation generally is studied.

**Table 4:** Studies categorization based on DNA methylation main use

Category	References
Prediction	[44–47, 55, 56, 58]
Monitoring	[41–44, 47–60]
Decision making/intervention	None

## Discussion

This systematic review on the application and value of DNA methylation as a biomarker in kidney transplantation shows heterogeneous and fragmented results. DNA methylation is a more accessible biomarker due to its low sensitivity to tissue handling compared with RNA or proteins and its analysis can even be performed on DNA isolated from small amounts of fixed tissue [63]. This biomarker could have an important role in different time points of the transplantation procedure and the subsequent short- and long-term follow-up. DNA methylation already proved, as other epigenetic mechanisms did, its role in the multiple biological events involved in post-transplant complications development [64, 65], with both the recipient and the donor organ continuously undergoing dynamic epigenetic modifications.

DNA methylation pattern alterations have already been associated in the past with IRI occurrence [23, 66–68]. The included studies showed how these alterations could be found in the urine of kidney transplant recipients [41] and could be related to perfusion state and DGF occurrence [43]. In the future, methylation assessment in different biological samples (kidney biopsy, urine, and blood) could be considered as an important early biomarker of acute kidney injury (AKI) during kidney transplantation, but further research is needed for a better understanding of its role in prediction, monitoring, and targeted therapy. Following the acute insult, fibrosis is the final common pathway of irreversible kidney transplant dysfunction. Its complex pathogenesis is triggered by an injury-induced fibroblast activation and matrix deposition that progresses even after the injury has disappeared. Evidence suggests that DNA methylation could in part be responsible for this process, acting through pro-fibrotic genes expression [7, 28, 69]. The included studies showed how DNA methylation pattern changes induced by oxidative stress and inflammatory setting could lead to this long-lasting fibroblast activation even in the context of kidney transplantation [42, 44] and how age-associated DNA methylation alterations at the time of the transplantation procedure could predict future injury [45]. Considering this, DNA methylation is a promising biomarker for the prediction of the development of chronic renal allograft dysfunction (CRAD) and could be used in the future for organ evaluation, prevention, and early intervention. As a consequence of this, preventing acute-injury-related DNA methylation alterations with modern preservation techniques could improve outcomes. The effect of normothermic machine perfusion on these biomarkers could be assessed and compared to that of static cold storage since this technique already proved to be able to recover previously discarded organs [70]. Moreover, these biomarkers could be used to optimize machine perfusion, acting on fluid compositions or operative parameters. Direct intervention on the markers through the perfusion fluid itself could also be an interesting opportunity in the future. The peculiar viable and isolated organ status typical of normothermic machine perfusion could allow the administration of demethylating agents and other additives while avoiding systemic side effects in the recipient. For a better understanding of the pathological processes leading to graft failure, and for an important role in monitoring and prediction of long-term complications, we recommend further investigations confirming the already established and promising associations, but also taking advantage of new high-resolution epigenome-wide DNA methylation assessment technologies to find new relevant loci and new associated patterns. The utilization of modern AI-based algorithms, for example, could help in integrating the massive amount of data provided by these new technologies to make accurate predictions, as will be described later.

DNA methylation could also have an important effect on long-term kidney function and development of fibrosis taking part in the recipient’s immune system modulation. DNA methylation is involved in the regulation of activation pathways [71], in the determination of cell plasticity [2], as well as in the control of the transcriptional profiles and functions of memory T cells [72] and NK cells [73–75] and therefore could influence the strength of the allospecific immune response. Various T cell populations are involved in this response and the included studies showed how DNA methylation pattern alterations occurred after kidney transplantation in PBMCs [58] and different T cell types [51], highlighting a possible involvement of DNA methylation in these processes even in the context of kidney transplantation. A better understanding of these dynamics could be useful in the future for the evaluation of the recipient response against the graft. Among CD4^+^ T cells, Treg cells are a CD4^+^ CD25^+^ T cell subset able to regulate inflammatory and immune responses [76, 77]. This subset was characterized by stable expression of *FOXP3*, a transcription factor that is essential for Treg cells function [78–80]. Stable expression of *FOXP3* is obtained thanks to DNA demethylation of *FOXP3* TSDR [81–84]. Treg cells can suppress the allograft-specific response using different mechanisms, ranging from suppressive cytokines to metabolite consumption [85]. Some of the included studies focused on the research of specific DNA methylation patterns associated with tolerance or other transplant-related conditions in order to understand them and to be able to predict long-term complications [47, 49, 52, 60]. These studies show how DNA methylation assessment might be crucial for Treg cell characterization and highlight the possible role of DNA methylation as a biomarker for post-transplant outcome prediction. A deeper understanding of the associations of specific DNA methylation patterns with post-transplant complications could also lead to the development of therapies based on epigenetic modifying agents.

Treg cells are central in the under-development tolerance-inducing cellular therapy. This therapy consists of the injection or implantation of living cells into a patient, with the potential to overcome the limitations of traditional drug treatment and to gain a deeper understanding of immune tolerance mechanisms [86]. Treg cell characterization through DNA methylation assessment is a crucial phase of these procedures, and TSDR methylation pattern has been studied in different kinds of patients to assess the suitability of these therapies. Kidney transplant recipients must undergo immunosuppression, a treatment that could impair Treg cell function as well as other immune cells function. For these reasons, the suitability of Treg cell-based therapy in transplant recipients has been studied by many researchers, focusing on the effect of different induction therapies [48] and different maintenance regimes [54, 57]. In one of the included studies, long-term treatment with belatacept showed better results in terms of the percentage of *FOXP3* demethylated cellular populations compared to other maintenance therapies [54]. However, the same research group proved later that these expanded cellular populations may require the use of epigenetic modifying agents to stabilize the TSDR demethylated status [57]. Further investigations are needed to understand the potential effect of the most commonly used medications, such as tacrolimus, prednisone, and basiliximab. Direct intervention on TSDR methylation status already proved to stabilize Treg cells for adoptive cell therapy [87, 88], but further studies are needed to clarify this aspect in the context of kidney transplantation and to improve current isolation and expansion techniques. Tolerance induction through Treg cells administration appears to be one of the most promising research topics trying to solve the organ deficiency problem and TSDR demethylation status assessment could be crucial for the characterization, *ex vivo* expansion, and stabilization of allospecific autologous Treg cell populations.

For the property of influencing and directing the immune response, DNA methylation appears also to be involved in the development of other complications than graft failure. Several studies focused on DNA methylation’s predictive value for complications such as cSCC [50, 55, 56] and infections [46], underlining the possible role of DNA methylation as a biomarker for other complications development and prediction. Although promising candidates with prognostic values significantly associated with survival and complications occurrence have already been identified, none of them is ready to be transferred into clinical practice because of the high heterogeneity of the studies [89].

Research in the field of kidney transplantation should be more focused on the predictive feature of DNA methylation modifications. Identifying patients at high risk for rejection or long-term complications through DNA methylation assessment would be a suited tool to guide clinical decision-making. As shown in Table 4, most of the research groups focused on prediction and monitoring, while currently, not a single study used DNA methylation for intervention or decision making. With the progress of tolerance induction therapy research, this could change, giving DNA methylation a central role in direct intervention and therapeutic strategies.

The growing interest for less invasive procedures to detect organ damage, the so-called liquid biopsies, raised the interest of the research community for DNA methylation analysis to quantify cell-free DNA (cfDNA). DNA methylation assessment is crucial for cfDNA origin identification [90]. After solid organ transplantation, donor-derived cfDNA (ddcfDNA) is released into the circulation, and the amount of ddcfDNA is representative of graft integrity. Dd-cfDNA can be distinguished from cfDNA originating from the recipient thanks to the genomic differences between donor and recipient typical of organ transplantation [91]. Nevertheless, tissue-specific DNA methylation patterns of cfDNA also provide the opportunity to identify the tissue origin of the detected genetic material [92]. An increase in ddcfDNA in blood plasma, either detected based on genomic differences or tissue-specific methylation patterns [93], has been reported to identify acute rejection [94, 95]. Moreover, methylated cfDNA in urine is one of the markers included in the Kidney Injury Test (KIT) to diagnose kidney injury as well as kidney allograft rejection [96, 97].

The problem of the heterogeneity of the studies concerning DNA methylation as a biomarker for kidney transplantation should be addressed. Designs of the included studies were mainly retrospective and covered mostly empirical evidence from case series. In consequence, the patient populations were also heterogeneous with a large variation in assessed epigenetic modifications, outcomes, and study designs. For what concerns phenotypes, more standardized definitions should be adopted. Allograft rejection, CRAD, IRI, kidney fibrosis, and DGF, for instance, are all phenotypes that can be defined with slight differences that can prevent comparison in a statistically valid meta-analysis. Despite common phenotype definitions, studies can also differ in other characteristics of the analyzed populations. For example, different duration of end-stage renal disease, time points of DNA methylation analysis, and purposes for DNA methylation analysis, albeit of potential scientific interest, are hampering the comparability of different studies in this early research phase. Analog considerations can be made for primary endpoints, with little agreement between study groups and the adoption of outcomes that might drastically complicate logistics, such as graft survival or recipient survival [98]. Moreover, the included studies adopted different DNA methylation analysis strategies, for example, different experimental approaches such as gene-specific or genome-wide analyses (summarized in Table 3) and even within similar experimental approaches, different technologies for DNA methylation analysis have been adopted. Furthermore, almost none of the studies report validation of their findings with another technique like pyrosequencing or biological validation as in variation in mRNA or protein expression.

Overall, the high number of confounders and the variety of arrays and protocols that have been used to assess common DNA methylation patterns prevent these studies from producing common statistically valid knowledge. An international clear consensus among the active research groups in this field should be discussed to designate important endpoints and produce more comparable results that could stimulate further research and generate new knowledge through the use of scientifically valid meta-analyses. Future studies should try to adopt prospective designs, with DNA methylation assessments performed before kidney transplantation and at specific time points after the procedure. Specific endpoints, DNA methylation analysis, and biological validation protocols should be adopted by different groups. This will not only enhance the comparability of these studies but will also lead to more cost-effective research. Here in this review, we provide insight on some of the previously discussed heterogeneities and on other potential implementations to help create a discussion that one day, hopefully, might lead to the development of DNA-methylation-based clinical tools to support decision-making in the kidney transplantation field.

In the last ten years, technology in the field of DNA methylation assessment quickly advanced, supported by the exponential growth of computational techniques for big data analysis. DNA methylation profiling techniques can be grouped based on the properties that are used to discriminate between methylated and unmethylated sites, namely enzyme digestion, affinity enrichment, and bisulfite conversion [99]. Due to their low resolution and to the quick development of bisulfite-conversion-based assays, enzyme-based (i.e., comprehensive high-throughput arrays for relative methylation, CHARM [100]) and affinity-based assays (i.e., methylated DNA immunoprecipitation, MeDIP [101]) are scarcely adopted in recent times and therefore will not be discussed in this manuscript [99]. In bisulfite-conversion-based methods, methylation-dependent changes are generated as bisulfite deaminates unmethylated cytosines into uracils, while methylated cytosines remain unchanged. These techniques, including methylation arrays, whole-genome bisulfite sequencing (WGBS), and reduced-representation bisulfite sequencing (RRBS), are characterized by single-base resolution and are among the most commonly used assays to study genome-wide methylation. Among methylation arrays, Illumina’s Infinium HumanMethylation450 BeadChip® (450 K array) and the updated version Illumina’s Methylation EPIC BeadChip® (EPIC array) are the most commonly used technologies to investigate the whole methylome [102]. These assays combine bisulfite conversion with amplification and hybridization of the converted DNA to arrays with predefined probes to assess the methylation status of around 450,000 and 850,000 methylation sites, respectively [103]. These technologies are characterized by high cost-effectiveness and by the need for low amounts of input DNA. However, their coverage is dependent on the array design, i.e., the selection of the predefined probes [99]. In WGBS, DNA is fragmented through sonication, repaired, added of an adenine base on the 3’ end, and successively ligated to methylated adapters. After size selection, bisulfite conversion is applied and the resulting genetic material is amplified and sequenced [104]. The great advantage of this technique is its ability to evaluate the methylation state of almost every CpG site in the genome. However, WGBS is expensive and impaired by DNA degradation after bisulfite treatment [99]. RRBS integrates restriction enzyme digestion, bisulfite conversion, and next-generation sequencing to analyze only specific fragments covering more than 85% of CpG islands while decreasing cost [105]. Nevertheless, RRBS focuses on promoters and areas close to the restriction site with low coverage of intragenic and distal regulatory elements [99]. Heterogeneous experimental approaches have been applied in the selected studies, introducing multiple confounders that impair comparability (summarized in Table 3). For instance, nine studies included in this review investigated DNA methylation on a whole-epigenome-scale all using bisulfite-conversion-based techniques, but the efficiency of bisulfite conversion was mostly not examined, while it represents a potential confounder. Methylation array was the most represented technique, accounting for eight out of nine studies, while only one study adopted WGBS. Considering the low cost and the need to increase the amount of available comparable data, we believe that the use of methylation arrays might be ideal in this early phase of research. Among methylation arrays, the 450 K array was the most common assay adopted in the studies included in this review while its updated version, the EPIC array, is being adopted by more and more research groups in recent times. The EPIC array contains over 850,000 probes, covering more than 90% of the probes of the 450 K array, but also covering sites in distal regulatory regions such as enhancer regions, overcoming one of the 450 K array’s main limitations [106]. Considering the better performances, the low price, and the compatibility with the 450 K array data shown by the very high per-sample correlation between the results of the two assays performed over the same samples [107], we recommend the use of the EPIC array for epigenome-wide DNA methylation assessment to take advantage of this solid technology as a research community, to produce comparable results, and to accelerate the process that might bring DNA methylation assessment into clinical practice.

After DNA methylation analysis, appropriate data analysis pipelines should be adopted to optimize the quality of the collected data. For instance, Wang et al*.* proposed an analysis framework for data collected with the 450 K arrays that, with the appropriate adaptations, is valid for the updated EPIC array [108]. Moreover, epigenetic data seems to be optimally suitable for machine learning, thanks to the stability over time of epigenetic modifications [109] and to the increasing availability of large-scale repositories [110–112]. As reviewed by Rauschert et al*.*, different machine learning methods have been applied to epigenomic datasets for the development of diagnostic systems, frequently using supervised learning methods [113]. Even though in the above-mentioned review the most adopted algorithm was random forest, we believe that deep learning algorithms [114] might have a big impact in the discovery of clinically useful epigenetic biomarkers for kidney transplantation thanks to the currently available high computational power and their capability to process highly dimensional datasets and identify complex patterns. However, we believe that a coordinated effort from the research community to make large epigenetic datasets publicly available and to elaborate standardized preprocessing pipelines is needed. These pipelines should include standardized normalization and imputation methods to increase data compatibility. These efforts would be crucial for a successful development and implementation of deep-learning-based tools in a clinical context.

**Fig. 3:**
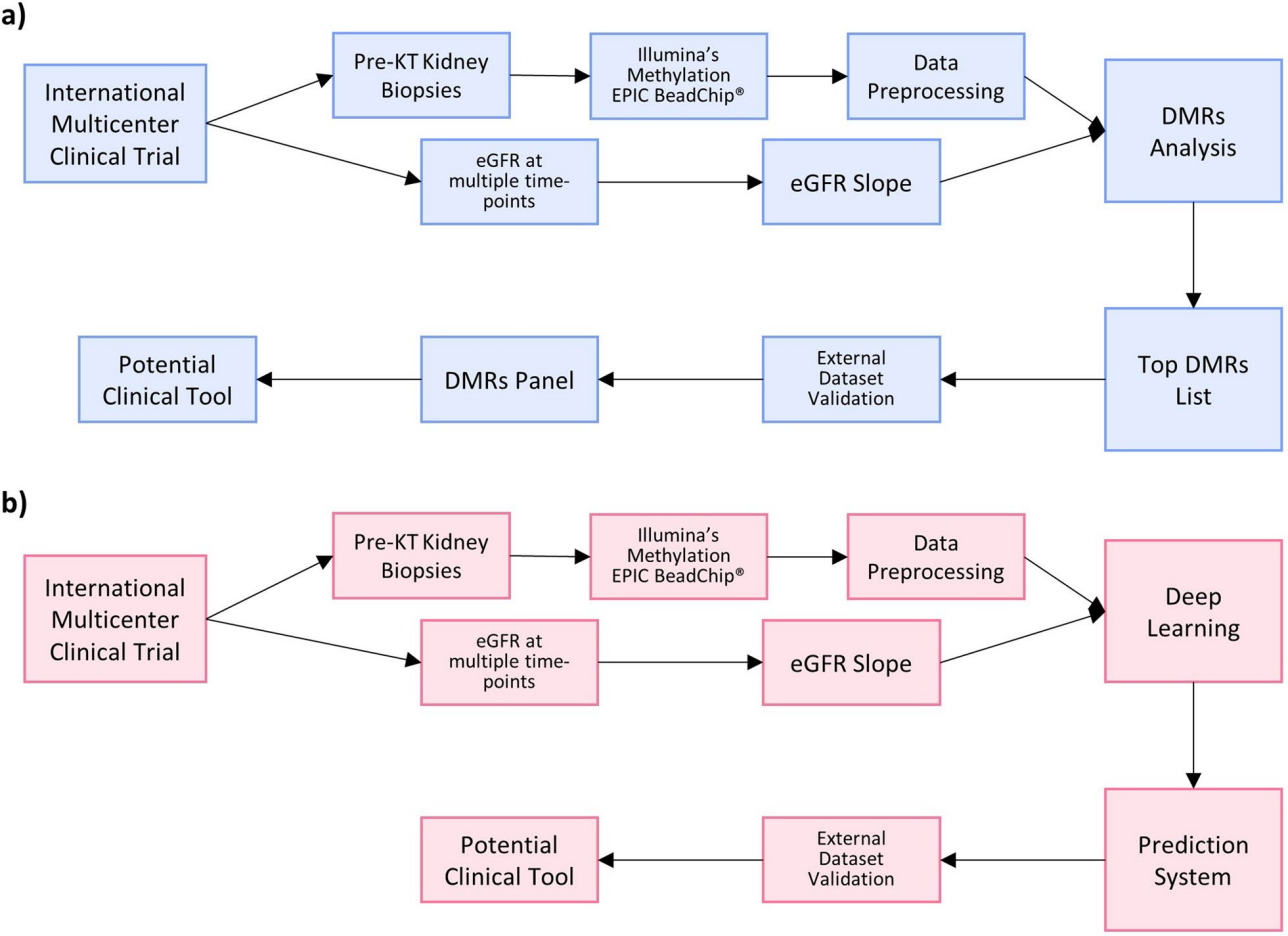
Overview of the proposed biomarker discovery pipelines. **a** Workflow of a proposed DNA methylation analysis pipeline for the discovery of differentially methylated regions associated with eGFR slope for the development of a limited panel that could be the base of a potential clinical tool. **b** Workflow of a proposed DNA methylation analysis pipeline for the development of a deep-learning-based prediction system. Abbreviations: DMR: Differentially methylated regions; eGFR: Estimated glomerular filtration rate; KT: Kidney transplantation

An ideal study setting for the discovery of clinically relevant biomarkers should collect data from a purposely designed multicenter international clinical trial. Since different DNA methylation patterns of multiple CpG sites between people of different ethnicity have been reported in the past [115], a high number of participants of different ethnicities and from different international centers would improve generalizability and tackle overfitting, one of the most common issues that accompany modern algorithms. In this early phase, analyzing pre-transplant kidney biopsies would be convenient considering the less invasive approach that minimizes the risk for complications. DNA methylation analysis should be performed on an epigenome-wide scale in order to identify new biomarkers that might associate with the outcome of interest. We believe that at this stage the research community should support the use of methylation arrays, i.e., the EPIC array, in order to produce comparable data with an accurate, widely available, and relatively cheap technology. Bisulfite conversion is a potential confounder, and its efficiency should be reported and taken into account during data analysis. For what concerns the investigated outcome, traditional clinical biomarkers of transplant outcome such as graft survival or recipient survival are characterized by low rates, requiring problematic sample sizes and follow-up periods. For this reason, the use of surrogate and composite endpoints, such as the estimated glomerular filtration rate (eGFR) slope, might improve data quality and speed up the implementation of DNA-methylation-based biomarkers into clinical practice [98, 116]. Data preprocessing should be handled in a standardized way, paying particular attention to quality control and within-array normalization. For this purpose, R [117] packages such as *Minfi* [112] and *methylumi* [118] might be the most suitable solution. Since the EPIC array uses two different types of probes, correction of probe design bias is also of uttermost importance. Multiple strategies have been elaborated to tackle this issue and solutions like Beta Mixture Quantile normalization (BMIQ) [119] or Regression on Correlated Probes (RCP) [120] should be appropriate. Two different approaches might then be followed (summarized in Fig. 3). A first approach (Fig. 3a) would be to use the normalized data to identify differentially methylated probes/differentially methylated regions using R packages like *Minfi*. Afterward, biological interpretation and gene ontology term enrichment analysis might then be performed with the R package *missMethyl* [121]. The detected differentially methylated regions might then be considered as candidate biomarkers for the selected outcome, and their association might be validated on other datasets. Validating these biomarkers might be of great value not only for monitoring and prognosis but also to better understand the processes underlying unfavorable post-transplant outcomes to elaborate novel therapeutic strategies. Successively, a panel composed of the eventually discovered biomarkers might be elaborated, leading to the development of easier and cheaper DNA methylation analysis pipelines of candidate genes based on bisulfite pyrosequencing that could enter the clinical practice. A second approach (Fig. 3b) would be based on the use of deep learning to produce surrogate endpoint predictions. Unfortunately, the most important features for these predictions would not be known, making the use of the whole EPIC array necessary even for the eventually developed clinical tool. However, the characteristics of epigenomic data, with the high number of probes and the multiple relationships between them, make it ideal to exploit the potential of deep learning to produce accurate predictions, making a big step toward the development of a clinical tool to support decision making. For that purpose, the use of the novel deep learning method *MethylNet* would be ideal to handle this type of data, considering its ability to construct embeddings, make predictions, and capture nonlinear interactions [122].

## Conclusions

DNA methylation is involved in acute ischemic injury, CRAD, and immune response modulation. So far, studies included in this review are heterogeneous in study design, DNA methylation analysis protocol, biological samples, and outcomes. DNA methylation analysis is increasingly being used in the field of kidney transplantation, but it is too early to affirm DNA methylation as a clinically relevant biomarker important for prevention, monitoring, and intervention. The studies described in this review highlighted its potential, especially considering the newest epigenome-wide methylation assessment technologies and novel discoveries in the field of big data analysis. An international agreement on study settings is needed to stimulate further research and achieve the first milestones in the quest for clinically useful biomarkers.

## Supplementary Information

**Additional file 1: Table S1**. Description of data: Search terms used for this systematic review.

**Additional file 2: Table S2**. Description of data: Risk of Bias assessment with the Newcastle–Ottawa scale for case–control studies.

**Additional file 3: Table S3**. Description of data: Risk of Bias assessment with the Newcastle–Ottawa scale for cohort studies.

**Additional file 4: Table S4**. Description of data: Risk of Bias assessment with the Newcastle–Ottawa scale for cross-sectional studies.

**Additional file 5: Table S5**. Description of data: Risk of Bias assessment with the Jadad scale for clinical trials.

## Data Availability

Not applicable.

## Abbreviations

450 K array: Illumina’s Infinium HumanMethylation450 BeadChip^®^
AKI: Acute kidney injury
BMIQ: Beta-mixture quantile normalization
CALCA: Calcitonin-related polypeptide alpha
CD: Cluster of differentiation
cfDNA: Cell-free DNA
CHARM: Comprehensive high-throughput arrays for relative methylation
CpG: Cytosine-phosphate-guanine
cfDNA: Cell-free DNA
CRAD: Chronic renal allograft dysfunction
cSCC: Cutaneous squamous-cell carcinoma
ddcfDNA: Donor-derived cell-free DNA
DGF: Delayed graft function
DNAmAge: Epigenetic age
eGFR: Estimated glomerular filtration rate
EPIC array: Illumina’s Methylation EPIC BeadChip^®^
FOXP3: Forkhead box P3 or scurfin
HLA: Human leukocyte antigen
IL: Interleukin
IRI: Ischemia–reperfusion injury
KIT: Kidney injury test
MeDIP: Methylated DNA immunoprecipitation
PBMCs: Peripheral blood mononuclear cells
RCP: Regression on correlated probes
RRBS: Reduced-representation bisulfite sequencing
Treg: Regulatory T
TSDR: Treg-specific demethylated region
WGBS: Whole-genome bisulfite sequencing
