# Supplementary material for: The applications of DNA methylation as a biomarker in kidney transplantation: a systematic review
Source: Clin Epigenetics. 2022 Feb 7;14:20. doi: 10.1186/s13148-022-01241-7 (PMC8822833; doi:10.1186/s13148-022-01241-7)
Supplement: Supplementary file 1 — Additional file 1: Table S1. Description of data: Search terms used for this systematic review. [file 13148_2022_1241_MOESM1_ESM.docx]

| **Embase.com (1971-): 3988** |
| --- |
| ('DNA methylation'/de OR 'DNA methyltransferase'/de OR 'DNA methyltransferase inhibitor'/exp OR 'methylome'/de OR 'DNA methylation assay'/de OR 'DNA demethylation'/de OR demethylation/de OR 'methylation'/de OR (((DNA OR region*) NEAR/3 (methyl* OR hypermethyl* OR hypomethyl* OR demethyl* OR dimethyl*)) OR methylat* OR methylome OR hypermethylat* OR hypomethylat* OR demethylat* OR dimethylat* OR azacitidin* OR decitabin* OR flucytosin*-deoxyribosid* OR fluorocyclopentenylcytosin* OR guadecitabin* OR phthaloyltryptophan* OR zebularin*):ab,ti) AND ('kidney transplantation'/exp OR 'organ transplantation'/de OR 'chronic kidney failure'/de OR 'end stage renal disease'/de OR 'kidney fibrosis'/exp OR 'graft recipient'/de OR 'kidney graft rejection'/de OR 'graft rejection'/de OR 'kidney failure'/de OR 'reperfusion injury'/de OR (((kidney* OR renal OR organ*) NEAR/6 (transplant* OR posttransplant* OR graft* OR allograft* OR allotransplant* OR fibros* OR recipient*)) OR (Graft* NEAR/3 (recipient* OR reject* OR delay*)) OR ((kidney OR renal) NEAR/3 (failure* OR ageing OR aging OR biological-age)) OR (reperfusion NEAR/3 (injury OR ischem* OR ischaem*)) OR ((chronic OR end-stage OR endstage) NEAR/3 (kidney OR renal) NEAR/3 (failure* OR disease* OR insufficien*)) OR ckd):ab,ti) AND [English]/lim. |
| **Medline ALL Ovid (1946-): 816** |
| (DNA Methylation / OR DNA Modification Methylases / OR DNA Demethylation / OR Demethylation / OR Methylation / OR (((DNA OR region*) ADJ3 (methyl* OR hypermethyl* OR hypomethyl* OR demethyl* OR dimethyl*)) OR methylat* OR methylome OR hypermethylat* OR hypomethylat* OR demethylat* OR dimethylat* OR azacitidin* OR decitabin* OR flucytosin*-deoxyribosid* OR fluorocyclopentenylcytosin* OR guadecitabin* OR phthaloyltryptophan* OR zebularin*).ab,ti.) AND (Kidney Transplantation/ OR Organ Transplantation/ OR Kidney Failure, Chronic/ OR Graft Rejection/ OR Renal Insufficiency/ OR Kidney Transplantation/ OR (((kidney* OR renal OR organ*) ADJ6 (transplant* OR posttransplant* OR graft* OR allograft* OR allotransplant* OR fibros* OR recipient*)) OR (Graft* ADJ3 (recipient* OR reject* OR delay*)) OR ((kidney OR renal) ADJ3 (failure* OR ageing OR aging OR biological-age)) OR (reperfusion ADJ3 (injury OR ischem* OR ischaem*)) OR ((chronic OR end-stage OR endstage) ADJ3 (kidney OR renal) ADJ3 (failure* OR disease* OR insufficien*)) OR ckd).ab,ti.) AND english.la. |
| **Web of Science Core Collection (1975-): 1119** |
| TS=(((((DNA OR region*) NEAR/2 (methyl* OR hypermethyl* OR hypomethyl* OR demethyl* OR dimethyl*)) OR methylat* OR methylome OR hypermethylat* OR hypomethylat* OR demethylat* OR dimethylat* OR azacitidin* OR decitabin* OR flucytosin*-deoxyribosid* OR fluorocyclopentenylcytosin* OR guadecitabin* OR phthaloyltryptophan* OR zebularin*)) AND ((((kidney* OR renal OR organ*) NEAR/5 (transplant* OR posttransplant* OR graft* OR allograft* OR allotransplant* OR fibros* OR recipient*)) OR (Graft* NEAR/2 (recipient* OR reject* OR delay*)) OR ((kidney OR renal) NEAR/2 (failure* OR ageing OR aging OR biological-age)) OR (reperfusion NEAR/2 (injury OR ischem* OR ischaem*)) OR ((chronic OR end-stage OR endstage) NEAR/2 (kidney OR renal) NEAR/2 (failure* OR disease* OR insufficien*)) OR ckd))) AND LA=(english) |
| **Cochrane CENTRAL register of trials (1992-): 27** |
| ((((DNA OR region*) NEAR/3 (methyl* OR hypermethyl* OR hypomethyl* OR demethyl* OR dimethyl*)) OR methylat* OR methylome OR hypermethylat* OR hypomethylat* OR demethylat* OR dimethylat* OR azacitidin* OR decitabin* OR flucytosin* NEXT deoxyribosid* OR fluorocyclopentenylcytosin* OR guadecitabin* OR phthaloyltryptophan* OR zebularin*):ab,ti) AND ((((kidney* OR renal OR organ*) NEAR/6 (transplant* OR posttransplant* OR graft* OR allograft* OR allotransplant* OR fibros* OR recipient*)) OR (Graft* NEAR/3 (recipient* OR reject* OR delay*)) OR ((kidney OR renal) NEAR/3 (failure* OR ageing OR aging OR biological-age)) OR (reperfusion NEAR/3 (injury OR ischem* OR ischaem*)) OR ((chronic OR end-stage OR endstage) NEAR/3 (kidney OR renal) NEAR/3 (failure* OR disease* OR insufficien*)) OR ckd):ab,ti) |
| **Google Scholar: 200** |
| "DNA methylation\|hypermethylation\|hypomethylation\|demethylation" "kidney\|renal transplantation\|graft\|allograft\|allotransplantation\|fibrosis\|recipient\|failure\|reperfusion\|failure\|insufficiency" |

**Additional file 1: Table S1** Search terms used for this systematic review.
