# Supplementary material for: The applications of DNA methylation as a biomarker in kidney transplantation: a systematic review
Source: Clin Epigenetics. 2022 Feb 7;14:20. doi: 10.1186/s13148-022-01241-7 (PMC8822833; doi:10.1186/s13148-022-01241-7)
Supplement: Supplementary file 2 — Additional file 2: Table S2. Description of data: Risk of Bias assessment with the Newcastle–Ottawa scale for case–control studies. [file 13148_2022_1241_MOESM2_ESM.docx]

**Additional file 2: Table S2** Risk of Bias assessment with the Newcastle-Ottawa scale for case-control studies.

| **Study ID** | **Newcastle Ottawa scale for case-control studies** | | | | | | | | | |
| --- | --- | --- | --- | --- | --- | --- | --- | --- | --- | --- |
|  | **Selection** | | | | **Comparability** | | **Exposure** | | | **Final score** |
|  | **Adequate case definition** | **Representativeness of the cases** | **Selection of controls** | **Definition of controls** | **Main factor** | **Additional factor** | **Ascertainment of exposure** | **Same method of ascertainment for cases and controls** | **Non-response rate** |  |
| Bestard 2011 [1] | * | * | * | 0 | * | * | * | * | 0 | 7/9 |
| Boer 2016 [2] | * | 0 | * | * | * | * | * | * | 0 | 7/9 |
| McGuinness 2018 [3] | * | 0 | * | 0 | * | * | * | * | 0 | 6/9 |
| Peters 2017 [4] | * | * | * | * | * | * | * | * | 0 | 8/9 |
| Peters 2019 [5] | * | 0 | * | * | * | * | * | * | 0 | 7/9 |
| Zhu 2020 [6] | * | 0 | * | * | * | * | * | * | 0 | 7/9 |

1. Bestard, O., et al., *Intragraft regulatory T cells in protocol biopsies retain Foxp3 demethylation and are protective biomarkers for kidney graft outcome.* Am J Transplant, 2011. **11**(10): p. 2162-2172.

2. Boer, K., et al., *Variations in DNA methylation of interferon gamma and programmed death 1 in allograft rejection after kidney transplantation.* Clinical epigenetics, 2016. **8**: p. 116-116.

3. McGuinness, D., et al., *A molecular signature for delayed graft function.* Aging Cell, 2018. **17**(5).

4. Peters, F.S., et al., *Differentially methylated regions in T cells identify kidney transplant patients at risk for de novo skin cancer.* Clin Epigenetics, 2018. **10**: p. 81.

5. Peters, F.S., et al., *Disrupted regulation of serpinB9 in circulating T cells is associated with an increased risk for post-transplant skin cancer.* Clin Exp Immunol, 2019. **197**(3): p. 341-351.

6. Zhu, C., et al., *DNA methylation modulates allograft survival and acute rejection after renal transplantation by regulating the mTOR pathway.* 2020. **n/a**(n/a).
