# Supplementary material for: The applications of DNA methylation as a biomarker in kidney transplantation: a systematic review
Source: Clin Epigenetics. 2022 Feb 7;14:20. doi: 10.1186/s13148-022-01241-7 (PMC8822833; doi:10.1186/s13148-022-01241-7)
Supplement: Supplementary file 3 — Additional file 3: Table S3. Description of data: Risk of Bias assessment with the Newcastle–Ottawa scale for cohort studies. [file 13148_2022_1241_MOESM3_ESM.docx]

**Additional file 3: Table S3** Risk of Bias assessment with the Newcastle-Ottawa scale for cohort studies.

| **Study ID** | **Newcastle Ottawa scale for cohort studies** | | | | | | | | | | |
| --- | --- | --- | --- | --- | --- | --- | --- | --- | --- | --- | --- |
|  | **Selection** | | | | **Comparability** | | **Outcome** | | | **Final score** |  |
|  | **Representativeness of the Exposed Cohort** | **Selection of the Non-Exposed Cohort** | **Ascertainment of Exposure** | **Demonstration That Outcome of Interest Was Not Present at Start of Study** | **Main factor** | **Additional Factor** | **Assessment of Outcome** | **Was Follow-Up Long Enough for Outcomes to Occur** | **Adequacy of Follow Up of Cohorts** |  |  |
| Bouvy 2014 [1] | * | * | * | * | * | * | * | * | * | 9/9 |  |
| Schaenman 2020 [2] | * | * | * | * | * | * | * | 0 | * | 8/9 |  |
| Soyoz 2021 [3] | * | * | * | * | 0 | 0 | * | * | * | 7/9 |  |

1. Bouvy, A.P., et al., *The impact of induction therapy on the homeostasis and function of regulatory T cells in kidney transplant patients.* Nephrol Dial Transplant, 2014. **29**(8): p. 1587-1597.

2. Schaenman, J., et al., *DNA Methylation Age Is More Closely Associated With Infection Risk Than Chronological Age in Kidney Transplant Recipients.* 2020. **6**(8): p. e576.

3. Soyoz, M., et al., *Consideration of IL-2, IFN-γ and IL-4 expression and methylation levels in CD4+ T cells as a predictor of rejection in kidney transplant.* Transplant Immunology, 2021. **68**: p. 101414.
