# Supplementary material for: The applications of DNA methylation as a biomarker in kidney transplantation: a systematic review
Source: Clin Epigenetics. 2022 Feb 7;14:20. doi: 10.1186/s13148-022-01241-7 (PMC8822833; doi:10.1186/s13148-022-01241-7)
Supplement: Supplementary file 4 — Additional file 4: Table S4. Description of data: Risk of Bias assessment with the Newcastle–Ottawa scale for cross-sectional studies. [file 13148_2022_1241_MOESM4_ESM.docx]

**Additional file 4: Table S4** Risk of Bias assessment with the Newcastle-Ottawa scale for cross-sectional studies.

| **Study ID** | **Newcastle Ottawa scale for cross-sectional studies** | | | | | | | | |
| --- | --- | --- | --- | --- | --- | --- | --- | --- | --- |
|  | **Selection** | | | | **Comparability** | | **Outcome** | | **Final score** |
|  | **Representativeness of the Sample** | **Sample size** | **Non-respondents** | **Ascertainment of the exposure (risk factor)** | **Main factor** | **Additional Factor** | **Assessment of Outcome** | **Statistical test** |  |
| Mehta 2006 [1] | * | 0 | 0 | ** | * | 0 | ** | * | 7/10 |
| Sherston 2014 [2] | * | 0 | 0 | ** | * | * | ** | * | 8/10 |
| Braza 2015 [3] | * | 0 | 0 | ** | * | * | ** | * | 8/10 |
| Trojan 2016 [4] | * | 0 | 0 | ** | 0 | 0 | ** | * | 6/10 |
| Trojan 2017 [5] | * | 0 | 0 | ** | 0 | 0 | ** | * | 6/10 |
| Heylen 2018 [6] | * | 0 | 0 | ** | * | * | ** | * | 8/10 |
| Bontha 2017 [7] | * | 0 | 0 | ** | 0 | 0 | ** | * | 6/10 |
| Heylen 2019 [8] | * | 0 | 0 | ** | * | * | ** | * | 8/10 |
| Rodriguez 2021 [9] | * | 0 | 0 | ** | * | * | ** | * | 8/10 |

1. Mehta, T.K., et al., *Quantitative Detection of Promoter Hypermethylation as a Biomarker of Acute Kidney Injury During Transplantation.* Transplant Proc, 2006. **38**(10): p. 3420-3426.

2. Sherston, S.N., et al., *Demethylation of the TSDR Is a Marker of Squamous Cell Carcinoma in Transplant Recipients.* 2014. **14**(11): p. 2617-2622.

3. Braza, F., et al., *Central role of CD45RA− Foxp3hi memory regulatory T cells in clinical kidney transplantation tolerance.* 2015. **26**(8): p. 1795-1805.

4. Trojan, K., et al., *IFNy + and IFNy − Treg subsets with stable and unstable Foxp3 expression in kidney transplant recipients with good long-term graft function.* Transplant Immunol, 2016. **39**: p. 1-9.

5. Trojan, K., et al., *Helios expression and Foxp3 TSDR methylation of IFNy+ and IFNy-Treg from kidney transplant recipients with good longterm graft function.* PLoS ONE, 2017. **12**(3).

6. Heylen, L., et al., *Ischemia-induced DNA hypermethylation during kidney transplant predicts chronic allograft injury.* J Am Soc Nephrol, 2018. **29**(5): p. 1566-1576.

7. Bontha, S.V., et al., *Effects of DNA Methylation on Progression to Interstitial Fibrosis and Tubular Atrophy in Renal Allograft Biopsies: A Multi-Omics Approach.* Am J Transplant, 2017. **17**(12): p. 3060-3075.

8. Heylen, L., et al., *Age-related changes in DNA methylation affect renal histology and post-transplant fibrosis.* Kidney Int, 2019. **96**(5): p. 1195-1204.

9. Rodriguez, R.M., et al., *Defining a Methylation Signature Associated With Operational Tolerance in Kidney Transplant Recipients.* Frontiers in Immunology, 2021. **12**(3352).
