# Supplementary material for: The applications of DNA methylation as a biomarker in kidney transplantation: a systematic review
Source: Clin Epigenetics. 2022 Feb 7;14:20. doi: 10.1186/s13148-022-01241-7 (PMC8822833; doi:10.1186/s13148-022-01241-7)
Supplement: Supplementary file 5 — Additional file 5: Table S5. Description of data: Risk of Bias assessment with the Jadad scale for clinical trials. [file 13148_2022_1241_MOESM5_ESM.docx]

| **Jadad scale for clinical trials** | | | | | | | | | |
| --- | --- | --- | --- | --- | --- | --- | --- | --- | --- |
| **Study ID** | **Randomization** | | **Blinding** | | **Withdrawals and dropouts** | **Inclusion/exclusion criteria** | **Adverse effects assessment** | **Statistical analysis** | **Total** |
|  | **Described as randomized** | **Appropriate randomization** | **Described as blinded** | **Appropriate blinding** |  |  |  |  |  |
| Alvarez Salazar 2017 [1] | * | * | * | * | * | * | * | * | 8/8 |
| Cortés - Hernández 2020 [2] | * | * | * | * | * | * | * | * | 8/8 |

**Additional file 5: Table S5** Risk of Bias assessment with the Jadad scale for clinical trials.

These two studies included patients participating in the clinical trial BENEFIT (IM103008 study) and we used information related to that study to evaluate risk of BIAS with this chart.

1. Alvarez Salazar, E.K., et al., *Methylation of FOXP3 TSDR Underlies the Impaired Suppressive Function of Tregs from Long-term Belatacept-Treated Kidney Transplant Patients.* Frontiers in Immunology, 2017. **8**(219).

2. Cortés-Hernández, A., et al., *Ex vivo expansion of regulatory T cells from long-term Belatacept-treated kidney transplant patients restores their phenotype and suppressive function but not their FOXP3 TSDR demethylation status.* Cell Immunol, 2020. **348**.
